# Supplementary material for: Screening, sorting, and the feedback cycles that imperil peer review
Source: PLoS Biol. 2026 Feb 24;24(2):e3003650. doi: 10.1371/journal.pbio.3003650 (PMC12931767; doi:10.1371/journal.pbio.3003650)
Supplement: S1 Appendix — (PDF) [file pbio.3003650.s001.pdf]

S1 Appendix: Mathematical proofs and additional results

A.1 Proofs

This section proves some of the main results for AO's model. The first two proofs below follow similar proofs found in AO [1], and are repeated here for completeness.

Claim. *The equilibrium $(\hat{q}, \hat{y})$ exists and is unique.*

Proof. Follows AO. Given a candidate marginal author $\tilde{q}$, author rationality pins down the marginal author's acceptance probability at c/v, which in turn pins down the journal's acceptance threshold $y(\tilde{q})$ and thus determines every other author's acceptance probability $\tilde{a}(q) = a(q, y(\tilde{q}))$. Let $\lambda(\tilde{q}) = \int_{\tilde{q}}^{1} \tilde{a}(q)\, dq$ be the volume of accepted manuscripts when the marginal author is $\tilde{q}$. Clearly, $\lambda(1) = 0$ and $\lambda' < 0$. Assuming $\lambda(0) \geq k$, there is then a unique solution to the capacity-filling condition $\lambda(q) = k$; this solution is $\hat{q}$ and the associated journal acceptance threshold is $\hat{y}$. In the unrealistic case that $\lambda(0) < k$, every author submits ($\hat{q} = 0$), the journal decreases $\hat{y}$ to whatever value fills its capacity, and every author has a strictly positive payoff. □

Claim. *The marginal author $\hat{q}$ decreases as review noise σ_Y increases.*

Proof. Follows AO. Let $\hat{q}$ and $\hat{a}(q)$ be the marginal author and acceptance function, respectively, with review noise σ_Y, and let $\hat{q}_1$ and $\hat{a}_1(q)$ give the marginal author and acceptance function under increased review noise $\sigma'_Y > \sigma_Y$. Suppose $\hat{q}_1 = \hat{q}$. Because the marginal author's acceptance probability is pinned down at c/v, AO use a result from Lehmann [2] to show that $\hat{a}_1(q) < \hat{a}(q)$ for all $q > \hat{q}$. Graphically, the acceptance probability curve under σ'_Y would rotate clockwise around the point $(\hat{q}, c/v)$. If every investigator $q > \hat{q}$ has a lower probability of acceptance, then the journal wouldn't fill its capacity. Hence thus the journal must lower its acceptance standard, increasing the acceptance probability of the previously marginal author $\hat{q}$ above c/v, thus making it worthwhile for more authors to submit papers. □

We make two notes about the proof above. First, as AO emphasize, the effect of increasing review noise on the journal's cutoff $\hat{y}$ is ambiguous. However, Lehmann's condition guarantees that, regardless of whether $\hat{y}$ increases or decreases, the acceptance probability $\hat{a}_1(q) < \hat{a}(q)$ for all $q > \hat{q}$, which drives the main result. Second, the applicability of Lehmann's condition depends on the details of the conditional distribution of Y given X (or alternatively the conditional distribution of Y given q). In our model, Lehmann's condition follows immediately from the fact that (X, Y) has a bivariate normal distribution (which itself follows from our assumption that the triple (θ, X, Y) has a trivariate normal distribution). As AO make clear, Lehmann's condition, and hence the above proof, holds more broadly; indeed, AO show that for Lehmann's condition to hold it suffices that the conditional distribution of Y given X has a location-scale structure $Y = X + \sigma_Y \epsilon$ for some random variable ϵ. However, Lehmann's condition does not necessarily hold universally for all distributions of (θ, X, Y). Articulating exactly the collection of distributions on (θ, X, Y) for which Lehmann's condition holds is beyond the scope of this article.

Claim. *The marginal author $\hat{q}$ decreases as v/c increases.*

Proof. Fix c. Let $\hat{q}$ and $\hat{y}$ give the equilibrium when the value to publication is v, and let $\hat{a}(q)$ give the associated acceptance function. Now consider the equilibrium when the value to publication is $v_1 > v$, and write the associated equilibrium quantities as $\hat{q}_1$, $\hat{y}_1$, and $\hat{a}_1(q)$. If $\hat{q}$ were to remain

the marginal author under v_1, then the acceptance threshold would need to be raised to $\tilde{y}_1 > \hat{y}$ to decrease $\hat{q}$'s acceptance probability to c/v_1. But an acceptance threshold of $\tilde{y}_1$ decreases the acceptance probability for every author $q > \hat{q}$, and thus the journal would no longer fill its capacity. Thus the journal threshold $\hat{y}_1$ must be $< \tilde{y}_1$, so $\hat{a}_1(\hat{q}) > c/v_1$, and hence the new marginal author $\hat{q}_1$ must be $< \hat{q}$. An identical argument holds when v is fixed and c decreases. □

A.2 Equations for model extensions

This section gives formal statements of several of the models in the main text. All models build from the AO model [1] as described in the main text.

A.2.1 Peer-review accuracy depends on reviewing load

Consider a single journal that sends every manuscript that it receives out for review. Let L denote the review load, such that if the marginal author is $\tilde{q}$, then $L = 1 - \tilde{q}$. Let the review noise σ_Y depend on L through the function $\sigma_Y = \Sigma_Y(L)$. We assume $\Sigma'_Y(L) \geq 0$. Write author q's acceptance probability when facing review threshold y with review noise σ_Y as $a(q; y, \sigma_Y) = \mathsf{Pr}\{Y \geq y | F_X(X) = q\}$ where $Y|q \sim \mathsf{N}(F_X^{-1}(q), \sigma_Y^2)$. Writing equilibrium acceptance probabilities as $\hat{a}(q) = a(q; \hat{y}, \sigma_Y = \Sigma_Y(1 - \hat{q}))$, the model equilibrium can again be found as the solution to AR and CF conditions:

$$v\,\hat{a}(\hat{q}) = c$$
$$\int_{\hat{q}}^{1} \hat{a}(q)\,dq = k.$$

The dependence of σ_Y on the review load is included via the new definition of $\hat{a}(q)$.

A.2.2 Single journal with desk review

Write author q's acceptance probability when facing desk-rejection threshold d and review threshold y with review noise σ_Y as $a(q; d, y, \sigma_Y) = \mathsf{Pr}\{D \geq d, Y \geq y | F_X(X) = q\}$ where the vector (D, Y) has conditional bivariate normal distribution

$$\begin{pmatrix} D \\ Y \end{pmatrix} \sim \mathsf{N}_2\left(\begin{pmatrix} x/(1+\sigma_X^2) \\ x/(1+\sigma_X^2) \end{pmatrix}, \frac{1}{1+\sigma_X^2} \begin{pmatrix} \sigma_X^2 + \sigma_D^2 + \sigma_X^2\sigma_D^2 & \sigma_X^2 \\ \sigma_X^2 & \sigma_X^2 + \sigma_Y^2 + \sigma_X^2\sigma_Y^2 \end{pmatrix} \right).$$

Continue to let the review noise σ_Y depend on L through the function $\sigma_Y = \Sigma_Y(L)$. Consider a candidate desk-rejection threshold d. Let $\hat{q}(d)$, $\hat{y}(d)$, and $\hat{\sigma}_Y(d) = \Sigma_Y(\hat{L}(d))$ be the marginal author, review threshold, and review noise induced by d, where $\hat{L}(d)$ is the review load induced by d and is given by

$$\hat{L}(d) = \int_{\hat{q}(d)}^{1} \mathsf{Pr}\{D \geq d | F_X(X) = q\}\,dq.$$

The journal's utility $u(d)$ is the average quality of accepted manuscripts, which writes as

$$u(d) = \frac{1}{k} \int_{\hat{q}(d)}^{1} \mathsf{E}\left[\theta | F_X(X) = q, D \geq d, Y \geq \hat{y}\right]\, a(q; d, \hat{y}(d), \hat{\sigma}_Y(d))\,dq.$$

This is identical to the expression for $u(d)$ that appears in the main text, with the sole exception that the notation has changed to make the dependence of the acceptance probability on $\hat{\sigma}_Y(d)$ explicit.

A.3 Additional results

A.3.1 Optimal blend of screening and sorting

S1 Figure shows the submission volume that maximizes the quality of published articles for a journal with capacity $k = 0.2$ as a function of the error in authors' private signals and the reviewers signals. The journal wants authors to be more (resp. less) selective about submitting their articles when authors' are better (resp. worse) judges of their manuscript's quality than reviewers. In other words, authors' partial revelation of their private information is more valuable to journals when authors are better than reviewers at assessing their manuscript's true quality, and vice versa. In S1 Figure, the volume of submissions $L = 1 - \hat{q}$ that maximizes the quality of published articles is shown as a function of the error in authors' (σ_X) and reviewers' (σ_Y) signals of a manuscript's quality. The journal's capacity is $k = 0.2$. Code to generate this Figure can be found in https://zenodo.org/records/15866736.

A.3.2 Computing reader welfare

Computing the reader welfare (or journal payoff) entails computing the mean of a truncated Gaussian distribution. For example, in the single-journal model without desk rejection, the average quality of a published article is

$$\mathsf{E}\left[\theta | F_X(X) \geq \hat{q}, Y > \hat{y}\right]. \tag{A.1}$$

This quantity is then standardized by the average quality of the top k articles to compute the reader welfare. Expectations of truncated multivariate Gaussian distributions such as expression A.1 were computed using the `mtmvnorm` routine from the `tmvtnorm` package in `R` [3].

To write the average quality of a published article for the model with several journals, continue with the notation developed there and write the equilibrium volume of manuscripts accepted on their jth submission when there are J total journals as

$$\lambda_J(j) = \int_{\hat{q}_j}^{1} b_j(q, \hat{y}_J)\, dq.$$

Of course, the capacity-filling condition requires $\sum_{j=1}^{J} \lambda_J(j) = k$. Write the average quality of manuscripts accepted on their jth submission as

$$\zeta_J(j) = \mathsf{E}\left[\theta | F_X(X) > \hat{q}_j, Y_1 < \hat{y}_J, \ldots, Y_{j-1} < \hat{y}_J, Y_j > \hat{y}_J\right].$$

The average quality of accepted manuscripts is just the weighted average of the $\zeta_J(j)$'s, using $\lambda_J(j)$'s as weights, which writes as

$$k^{-1} \sum_{j=1}^{J} \zeta_J(j) \lambda_J(j).$$

A.3.3 Underuse of desk rejection from shared reliance on a common reviewer pool

Here, we present a separate model that demonstrates how competition between journals results in an underuse of desk-rejection and overexploitation of the reviewer pool. This model and its underlying logic are essentially identical to the classic Cournot oligopoly model in economics [4]. This model stands on its own and does not inherit any notation or assumptions from the model presented in the main text.

Ignore screening. Suppose that, of the manuscripts submitted to a journal, a fraction $\rho \in [0,1]$ are suitable for publication. Let $\theta \in \{0,1\}$ code for a manuscript's suitability, with $\theta = 1$ indicating that a manuscript is suitable for publication. For every suitable manuscript that a journal publishes, it receives a payoff $v_1 > 0$, and for every unsuitable manuscript that it publishes, it receives the negative payoff $v_0 < 0$. The journal receives a payoff of 0 for rejecting a manuscript. The fraction of suitable manuscripts is sufficiently low that a journal prefers to reject every manuscript instead of publishing every manuscript, $v_0(1-\rho) + v_1\rho < 0$.

A journal must decide which fraction of its manuscripts to send out for review and which fraction to desk-reject. Let $L \in [0,1]$ be the fraction of manuscripts sent out for review, or the review load. If a manuscript is sent out for review, it is evaluated by a single reviewer who generates a binary report $Y \in \{0,1\}$, where $Y = 1$ if the reviewer reports that the manuscript is publishable and $Y = 0$ otherwise. Each reviewer is characterized by a pair of error rates (α, β), where $\alpha = \mathsf{Pr}\{Y = 1|\theta = 0\}$ is the conditional probability that a reviewer incorrectly reports that a manuscript should be published when it is unsuitable, and $\beta = \mathsf{Pr}\{Y = 0|\theta = 1\}$ is the conditional probability of a report that a suitable manuscript should not be published.

If a journal sends a manuscript out for review, it must act in accord with the reviewer's report. If the reviewer reports $Y = 1$, the manuscript is published, otherwise the manuscript is rejected. The value to the journal of sending a manuscript to a reviewer with accuracy (α, β) is

$$\begin{aligned} v(\alpha, \beta) &= v_1 \mathsf{Pr}\{\theta = 1, Y = 1\} + v_0 \mathsf{Pr}\{\theta = 0, Y = 1\} \\ &= v_1(1-\beta)\rho + v_0\alpha(1-\rho). \end{aligned}$$

Note that the journal prefers to desk reject a manuscript instead of sending it to a reviewer whose report is independent of the manuscript's suitability (i.e., $v(\alpha, 1-\alpha) < 0$ for any α); the journal prefers to send a manuscript to a perfectly accurate reviewer instead of desk rejecting it ($v(0,0) > 0$), and the journal is better off when the reviewer is more accurate (both $\partial v/\partial\alpha < 0$ and $\partial v/\partial\beta < 0$). Because a journal's payoff will be based only on $v(\alpha, \beta)$ and not α and β directly, label reviewers by the expected value that they generate for the journal, $v = v(\alpha, \beta)$, i.e., "reviewer v", and call v the reviewer's value.

Suppose reviewers differ in their value, and that the distribution of values among reviewers is continuously distributed on an interval contained in $[v_0(1-\rho), v_1\rho]$. Let $v(q)$ be the value of the reviewer at the $(1-q)$th quantile, such that $v(0)$ is the value of the best reviewer and $v(1)$ is the value of the worst, with $dv/dq < 0$. A journal's payoff to sending out L manuscripts for review and desk-rejecting the rest is

$$\pi(L) = \int_0^L v(q)\, dq = L\bar{v}(L)$$

where $\bar{v}(L) = L^{-1} \int_0^L v(q)\, dq$ is the average value of the top L reviewers. To maximize its payoff, the journal chooses L to satisfy the first-order condition $\pi'(L) = v(L) = 0$. Let L^* denote this optimal load. In other words, the journal solicits reviews from all reviewers with $v \geq 0$, and then desk-rejects the remaining manuscripts.

Now suppose there are J competing journals that all rely on the same common pool of reviewers. Each journal $j = 1, \ldots, J$ receives a randomly selected proportion $1/J$ of the total manuscripts, and must decide what fraction $L_j \in [0, 1/J]$ to send out for review. If a journal decides to send a manuscript for review, it does not necessarily receive its first choice of reviewers. Instead, if together the J journals send a total of L manuscripts out for review, then each journal obtains its reviews from a random subset of the L best reviewers. Let L_j be the fraction of its manuscripts

that journal j sends out for review, and let L_{-j} give total volume of manuscripts sent out for review by the other $J-1$ journals. The payoff to journal j is

$$\pi_j(L_j, L_{-j}) = \frac{L_j}{L} \int_0^L v(q)\,dq.$$

We seek a symmetric, pure-strategy Nash equilibrium. Let L_j^* give each and every journal's equilibrium action, and let $L_J^* = J \times L_j^*$ give the total review load at this equilibrium. Solving the first order conditions $\partial \pi_j / \partial L_j = 0$ and seeking the symmetric solution in which $L_{-j}^* = (J-1) \times L_j^*$ shows that L_J^* solves

$$v(L_J^*) + (J-1)\bar{v}(L_J^*) = 0. \tag{A.2}$$

Because $v(L) < \bar{v}(L)$ for all L, it follows that L_J^* is increasing in J (and strictly increasing as long as there is an internal equilibrium.)

Thus, as J increases, journals desk reject manuscripts more sparingly and send more manuscripts out for review. More precisely, as J gets very large, the total review load shifts from one in which the marginal reviewer makes the journal no better off than desk rejection ($v(L_1^*) = 0$) and approaches one in which the average reviewer makes the journal no better off than desk rejection ($\lim_{J\to\infty} \bar{v}(L_J^*) = 0$).

Cited Literature

[1] Jérôme Adda and Marco Ottaviani. "Grantmaking, grading on a curve, and the paradox of relative evaluation in nonmarkets". In: The Quarterly Journal of Economics 139.2 (2024), pp. 1255–1319.

[2] E. L. Lehmann. "Comparing location experiments". In: The Annals of Statistics 16.2 (1988), pp. 521–533.

[3] Stefan Wilhelm and Manjunath B G. tmvtnorm: Truncated Multivariate Normal and Student t Distribution. R package version 1.6. 2023.

[4] Augustin Cournot. "Recherches sur les Principes Mathématiques de la Théorie des Richesses". In: English edition: Researches into the Mathematical Princples of the Theory of Wealth (1897). Ed. by N. Bacon. London: Macmillan, 1838.
