# Supplementary figures and images for: Screening, sorting, and the feedback cycles that imperil peer review
Source: PLoS Biol. 2026 Feb 24;24(2):e3003650. doi: 10.1371/journal.pbio.3003650 (PMC12931767; doi:10.1371/journal.pbio.3003650)

Reviewer noise,  $\sigma_Y$

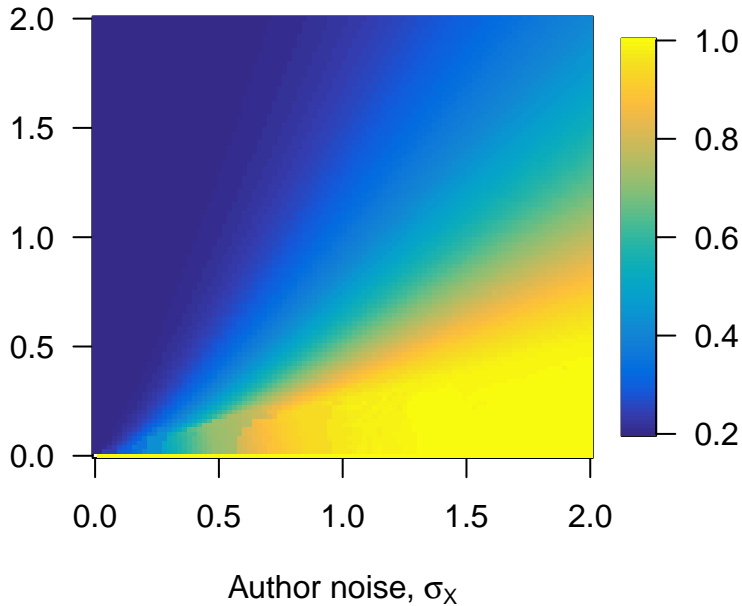

Supplement: S1 Fig — (PDF) [file pbio.3003650.s002.pdf]
