# Supplementary material for: Screening, sorting, and the feedback cycles that imperil peer review
Source: PLoS Biol. 2026 Feb 24;24(2):e3003650. doi: 10.1371/journal.pbio.3003650 (PMC12931767; doi:10.1371/journal.pbio.3003650)
Supplement: Attachment — Submitted filename: BergstromGross_Responses.pdf [file pbio.3003650.s003.pdf]

Point-by-point responses

At the outset, we want to respond to the important point raised by the Academic Editor as well as several of the Reviewers. In his cover letter, Dr. Roberts writes:

> *"I should also say that before review the Academic Editor had expressed some concerns somewhat similar to reviewer 3's final point: 'Among possible remedies, they fail to mention the most obvious one: reducing the weight given to the number of publications and their JIF in assessing researchers. Nor do they discuss distributed review. The paper is elegantly written, and I agree the topic is of great interest to both researchers and publishers. But it offers neither group a practical way forward—especially if the arguments for particular remedies rest on equations that few people understand and even fewer could test.' We think further consideration should be given to this."*

Similar sentiments are expressed in the closing comments of Reviewer 2, notwithstanding the mention of distributed peer review (which we discuss below). In response, we have added a new paragraph at the end of the Discussion that we hope captures the spirit of these suggestions. There, we argue that decreasing the value attached to publication (or v, in our model) is not so simple, because v is set by the scientific community, and thus cannot be changed unilaterally by a single actor. Further, v serves a variety of other functions in science, such as motivating researchers to work hard and to take scientific risks (as we argued in our 2024 *PLOS Biology* paper). Nevertheless, both the descriptive and normative questions about why the scientific community rewards scientific activities as it does remain open and are made more urgent by the pressure on peer review that large v creates.

Respectfully, distributed peer review (as we understand it) seems to us to fall outside the scope of our manuscript. While we are intrigued by the broader idea of attaching authors' opportunity to publish with their service as a reviewer, formally contemplating distributed peer review—or any form of obligatory peer review—would require endogenizing reviewer effort explicitly. Currently, our model implicitly endogenizes reviewer effort by citing it as one mechanism that causes review accuracy to decline as the review load increases. However, if we add the additional dimension of voluntary vs. obligatory peer review, we would need to explicitly account for the drop in review effort that would likely ensue with obligatory review. Computer-science conferences provide a cautionary tale here, as they rely on distributed peer review and now seem to be dealing with a crisis of both AI-generated reviews and manuscripts strategically written to survive AI-generated review (see ref. 32). To accommodate this properly in our model, we would need to explicitly account for both the drop in reviewer effort that seems to ensue from obligatory peer review and authors' strategic responses to this drop. All in all, we prefer not to add this layer to the model, but we would be happy to reconsider if the Editor wishes.

Detailed responses to individual Reviewer comments follow. We thank each Reviewer for their variety of constructive comments. For each Reviewer, we begin our responses with the portion of the review that suggests actionable responses.

Reviewer 1

R1-1. *The authors explicitly state that: "Vertical" proliferation, in which journals more densely occupy niches along a prestige spectrum, is outside the present scope (but see ref. [38]). I accept this choice even though my lab's research demonstrated the existence of such vertical integration (Stringer et al PLOS One and JASIST).*

Response: To clarify, we don't question the existence of vertically differentiated journals. Our model just can't handle the complexity that vertical differentiation between selective journals that induces. We have added text to explain why vertical proliferation lies outside our present scope (line 260 in the revision without tracked changes). In short, vertical proliferation makes the author's action space substantially larger, which in turn makes their strategy space (a strategy being a sequence of actions) even larger yet.

R1-2. *Nonetheless there is an interesting mechanism that is not explored but is definitely important. Again, this is inspired by my lab's research (Moreira et al PLOS One). It seems plausible that authors have a specific* $\bar{q}$*. That is, a manuscript i produced by author j does not have* q_j^i *drawn from* $N(0, \sigma_x)$ *but instead from* $N(\bar{q_j}, f * \sigma_x)$ *where* $f < 1$*. This fact may not appear to be important for the default case but I think it is important for the desk review case. While the editor's* σ_D *may be large, one could imagine that over time an editor will learn to recognize high* $\bar{q_j}$ *authors and be guided by it for deciding on desk rejection. I believe that an exploration of the impact of such a mechanism on desk rejections in terms of societal welfare would be important, especially in terms of exclusion of high* $\bar{q_j}$ *authors early in their career and slacking of high* $\bar{q_j}$ *authors late in their career.*

Response: It seems to us that there are several intriguing ideas included in this comment. To clarify, in our model an author's θ is not necessarily a random draw from a $\mathcal{N}(0, 1)$ distribution. Instead, the standard normal is merely the marginal distribution of θ across all authors. It may well be that there are covariates (such as author identity) that are correlated with θ, and hence are also correlated with the author's signal X and the journal's signal Y. To the extent that these covariates are known to both the author and the journal, knowledge of these covariates decreases both the noise in the author's signal σ_X and the noise in the journal's signal σ_Y. To this extent, our model already captures the effect of covariates that authors and journals might use as surrogates for article quality, such as author reputation.

However, this comment highlights further considerations that our model does not engage. First, author identity might be a weaker predictor of θ for early-career authors than for late-career authors. In the model, this would manifest as both σ_X and σ_Y varying among authors. Heterogeneity in σ_X and σ_Y is beyond the scope of our model, but surely impacts author behavior. Indeed, the effects of author reputation would seem to be tightly connected to the fact that junior authors often recruit senior authors onto their papers.

Second, the comment also suggests that senior authors might coast on their reputation late in their careers. This is a juicy suggestion, but it seems to us that reality is more complex. On the one hand, while established authors might try to coast on their reputation late in their

careers, journals and readers aren't naive. If an established author slacks—or if established authors in general establish a reputation for slacking—then journals and readers will learn this and will discount the work of established authors accordingly, albeit perhaps with a lag. Second, if journals do give established authors the benefit of the doubt, they only do so because readers do the same. Journals can hardly be blamed if busy readers gravitate to papers written by established authors. Finally, taking the longer view, early- and mid-career scientists are extraordinarily motivated to build a reputation for doing good work. Whether or not these incentives serve science well is far beyond anything we can answer here, but it at least seems plausible that the net effect of reputational considerations is positive, even despite the prospect of late-career coasting.

In any case, all of this is to say that these considerations are fascinating but also multifaceted and complex, and thus beyond anything that we can satisfactorily address in this paper.

Reviewer 2

R2-1. *The model is deeply rooted in normal distributions: the manuscript quality, the errors in evaluating the quality by both authors and reviewers are all Gaussian. While this makes a lot of sense when modeling errors, other quantities (esp. manuscript quality) could have very skewed distributions. For example, the number of citations, which can be considered a first-order estimate of readership, tends to be lognormally distributed. If I am not mistaken, changing the distribution of manuscript quality would have absolutely no effect on the conclusions (because only the percentile of quality matters). If this is the case, and whenever this is the case, the Authors could add a line saying that these assumptions do not matter (at least qualitatively). In a similar vein, one drawback of Gaussians is that the error functions cannot be expressed algebraically, and as such for example the existence of an equilibrium can be proven, but cannot be written explicitly in closed-form using the model parameters. Would the choice of different distributions alleviate this issue? If one had an explicit formula for $\hat{q}$ and $\hat{y}$ in terms of v/c and the various standard deviation, it would be very easy to show the effect on welfare etc by taking derivatives.*

Response: We agree that the assumption of normality throughout limits the model, and we also agree that assuming normality for θ is the most open for debate. Indeed, some of Reviewer 1's work shows that the distribution of citation counts is log-normal, although we might argue that θ in our model, and scientific import more generally, is not exactly the same as—and thus not necessarily as right-skewed as—an article's citation count.

Regardless, while we suspect that our qualitative results are robust to plausible departures from normality, it is not easy to relax the assumption of normality in the present context, for two reasons. First, AO's result—that an increase in review noise increases the volume of submissions, and vice versa—does not obviously extend to alternate distributions for θ. To be clear, AO's result *may* apply for a larger collection of distributions on θ, and we speculate that it does, but a proof of how far AO's result goes currently eludes us. Our restatement of AO's proof in the appendix did not make this clear, and we have added a new paragraph

to clarify. For what it's worth, AO extended their results to a broader set of distributions on θ by assuming that authors know their manuscript's quality exactly ($\sigma_X = 0$). We could extend our results along the same lines, but imperfections in the authors' signals are an important part of our story, especially in the multi-journal case. We think that assuming normally distributed θ is more viable than assuming that authors know their manuscript's quality exactly.

Second, the assumption of normality eases the computations throughout. Specifically, conditional expectations like $\mathsf{E}[\theta | F_X(X) \geq \hat{q}, Y > \hat{y}]$ that appear in the reader's welfare (and the journal utility in the desk-rejection model) and the acceptance probabilities $a_j(q; y)$ and $b_j(q; y)$ in the multi-journal model can be computed using standard results when everything is normally distributed. If we relax the assumption of normality on θ, all these computations become numerical integrations which get nasty quickly. We'd love to be able to find alternative choices for the distribution of θ which would make these computations equally straightforward, but we don't know of any choices that would.

R2-2. *The analysis is very much based on equilibria: the existence of critical values $\hat{q}$ and $\hat{y}$ is proven, and then these values determine the welfare for authors, readers and reviewers. While reading the manuscript I kept thinking that a) authors have but a vague idea of $\hat{y}$, and b) journals also have only a rough estimate of $\hat{q}$. In reality, both authors and journals constantly adjust their submission/acceptance criteria based on a rapidly-shifting landscape, the birth/death of journals, etc. The analysis, as it stands, reminds me of classic game theory: the authors try to maximize their payoff, and journals to fill their pages with the best manuscripts, all seen as a "static game". I imagine that the same could be turned into an "evolutionary game", in which journals and authors correct their course in time, as a result of previous outcomes. A dynamic version of the model would also allow to study the relaxation to equilibrium following a perturbation (e.g., the cited increase in submissions following the pandemic), as well as the study of "interventions" on the parameters of the game. Right now, a dynamic version is mentioned in passing (l236), but in my opinion it would be an exciting avenue for the future.*

Response: We agree that a dynamic or evolutionary game would be an exciting future direction. Adding such a game to this article would ba a major undertaking, however. We prefer to leave it for a future effort.

R2-3. *In terms of reflections, I appreciated the thoughtful discussion, which is very much centered on the cost-side (c) and on increasing the supply of reviewers (for example by paying them). One aspect that I did not find discussed, but that greatly impressed me over the past 20 years, is the rise of bean-counting institutions (funding agencies, tenure committees etc) that want to measure the value (v) discussed in the manuscript directly (as impact factor, classification of journals into tiers, etc), and use it for real-world decisions. Even though we know that all Deans can count, but few can read, scientists have a role to play in reducing the differential value of publication venues for careers, and increase the weight attributed to the quality of the work (as opposed the impact factor of the journal in which it was published).*

For example, my institution limits the number of publications that can be submitted in a tenure/promotion package to five, thereby incentivizing researchers to concentrate on the quality of their work, rather than the number of papers published.

Response: Please see our response to the Editor at the beginning of this document. We applaud the Reviewer's institution for the steps taken there to de-emphasize publication quantity at tenure and promotion time. One of us (KG) is currently arguing for similar steps at our own institution, and appreciates all the peer pressure that can be brought to bear on that case. For what it's worth, however, it seems to us that the biggest career bottleneck in academics is getting a job in the first place, and the sense persists (whether accurate or not) that getting a job at a premier institution requires publications in top outlets (see ref. 47).

<u>Reviewer 3</u>

R3-1. *In its present state, I think there is a danger that many readers seeing the simplifying assumptions of the models might too quickly disregard the insights they have to offer, so some additional effort should be made in the presentation of the work, both to explain the workings of the model to those less conversant with the mathematical techniques used and to emphasise how, despite the simplifying assumptions, the model relates to the real world of modern day scholarly publishing. I have some specific comments along these lines that, if addressed, may give the paper greater impact with its intended audience. They are a mixture of major and minor comments. I will lay them out in the order that they occur in the manuscript.*

Response: We thank the Reviewer for this constructive feedback. We address the Reviewer's specific points below.

R3-2. *Line 2 - a minor point but the claim that publishers rely on peer review to identify 'the best science' is over-simplistic and starts the paper off, if not on the wrong foot, then with a stumble. There are countless mid and lower tier journals where manuscripts reporting perfectly decent science are submitted but which neither the authors not the reviewers would consider 'the best science'. The best submissions for a given journal might be a better description; or perhaps it could be claimed that peer review strives to provide a valuable quality check on submitted manuscripts (its well-known weaknesses notwithstanding).*

Response: Here and throughout, we intended the phrase "the best science" to mean "the best science among the papers submitted to a given journal", as the Reviewer suggests. It seems to us that while the additional qualification adds precision, it is also wordy. We have changed the first sentence of the abstract to read "Scholarly journals rely on peer review to identify the science most worthy of publication." Elsewhere (later in the abstract, and in the third paragraph of the Introduction), we have retained the phrasing "the best science". We think readers will understand that this means the best science among the papers submitted to a journal, but we are happy to reconsider if the Editor feels differently.

R3-3. *Line 27 - another minor point but I doubt that many authors have 'full awareness' of the evaluation to follow. I would qualify slightly - probably they have a reasonable idea of the standard expected of their journal of choice.*

Response: We agree that "full awareness" was too strong. We have changed the text to read "in anticipation of the evaluation to follow".

R3-4. *Line 62 - this reader (as I suspect many other readers of PLoS Biology) is not familiar with the literature on agent-based models. It would be helpful to have a brief explanation of what these are and how they differ from the approach taken by the authors. I was left wondering why agent-based models were unable to unearth the feedback mechanisms that are a key - novel? - feature of Bergstrom & Gross's model.*

Response: We have changed "detailed agent-based models" to read "detailed, agent-based simulation models", and also changed the following sentence to read "we (develop) ... low-dimensional models that isolate how peer review shifts the burden..." in hopes of clarifying the methodological distinction between our approach and previous work. We're reluctant to go more deeply into the pros and cons of detailed simulations vs. analytical models more fully here, because the complementary strengths and weaknesses of these approaches have been discussed at length elsewhere and nothing about the context of peer-review adds new wrinkles to that discussion. Nonetheless, if the Editor feels more detail would be helpful, we would be happy to add it.

R3-5. *Page 4 - Fig 1 - The legend refers to screening but not sorting. While these terms are defined implicitly in the body text, it would be useful to give an explicit definition and use the terms consistently throughout.*

Response: We thank the Reviewer for pointing this out. We have revised Figure 1 so that the word "sorting" appears in the flow diagram, and we have changed the legend of Figure 1 to more explicitly define the relationships among screening, sorting, and peer review.

R3-6. *Pages 4-6 - the description of the mathematical model is reasonably clear but would benefit from some additional explanation for a wider readership. Line 88 - 'journal capacity' is pretty self-evident but the reader will have to work less hard if it is also stated that this means the total number of articles published (per issue or per year).*

Response: We have rephrased the paragraph in question to clarify that the capacity k is a proportion and not a count. It is important that k is a proportion instead of a count, because the strength of competition among authors is determined by the proportion of authors that the elite journal can publish, as opposed to the number of manuscripts that the elite journal can publish. Our previous text did not make it immediately clear that k was a proportion, and we thank the Reviewer for catching this.

R3-7. *Line 93 - explain why it helps to suppose that there is a 'unit mass of authors'.*

Response: This is a good point. There are two important consequences of assuming a unit mass of authors. First, we don't have to explicitly account for the size of the community, as long as the community is large enough to justify the "unit mass" assumption. Second, we don't have to worry about discreteness considerations that would arise if we did consider the size of the community explicitly. We have added a parenthetical statement following the phrase in question to clarify.

R3-8. *Line 94 - why 'is endowed with'; why not the simpler 'has'?*

Response: We appreciate the Reviewer's point, but, respectfully, we think that the phrase "is endowed with" has a useful richness here that "has" lacks. In particular, "is endowed with" emphasizes that the model begins at the instant when authors hold fully formed manuscripts and must decide whether or not to submit them. In turn, this foreshadows one of the model limitations that we discuss later, namely, that in reality authors are not endowed with fully formed manuscripts but instead must decide how much effort to invest in developing them, which is also a decision made in anticipation of the how the manuscript will fare in review. The simpler "has" doesn't plant that seed as effectively.

R3-9. *Lines 96 and 98 mention the authors "own sense of whether their work is any good" and "their private signal X" of their manuscript's quality; wouldn't it be better not to have two different descriptions of the same thing?*

Response: We have modified the text here so that X is defined directly as the author's sense of their work's quality.

R3-10. *Line 100 - explain what is meant by 'quantile'.*

Response: Respectfully, we think 'quantile' is a common term that should be available to readers of PLOS Biology. We debated whether we should convert "quantile" to the perhaps more familiar "percentile", but the conversion to percentiles requires the awkward step of multiplying q by 100%, which seems unnecessarily cumbersome.

R3-11. *Line 120 - I did not understand the sentence in parenthesis about indifferent authors.*

Response: We have changed this parenthetical comment to read: "Authors on the razor's edge of indifference—those for whom the benefit from submitting their manuscript exactly matches the cost—are rare enough that their behavior does not affect the model equilibrium."

R3-12. *Page 7 - Fig. 2 - I think these graphs would be clearer if they had more annotation (to reduce the need to consult the legend); e.g. add the values of sigma or v/c to the plot ((e.g. in panel A, add v/c = 5 etc). If panels B & C could be side by side, that would*

facilitate the comparison discussed in the text.

Response: Respectfully, it seems to us that additional annotation within the graphic will clutter the figure. We appreciate that reasonable minds may disagree here, but we prefer to keep the figure annotated as is. We have changed the arrangement of the panels so that panels B and C are now side by side, as per the Reviewer's suggestion.

R3-13. *Line 159 - In addition to presenting the expression for authors' welfare, I would suggest saying in words that the welfare diminishes with c and L, and increases with c and k - just for maximum explicitness.*

Response: Respectfully, we don't think this is necessary. We'd like to keep the manuscript moving here, and reiterating these relationships as the Reviewer suggests slows the momentum down without adding anything new.

R3-14. *Line 165 - the statement "The readers' welfare measures how reliably publication indicates scientific quality" is indicative of the simplicity of the model since, arguably, the quality of the manuscripts in any given journal takes a range of values. Perhaps some qualification of the statement could be offered or a less definitive verb than "measures" could be used? "Estimates"?*

Response: We take the Reviewer's point that our previous sentence was poorly worded; we conflated the readers' welfare with our measure of it. We have rewritten the paragraph in question to be more precise.

R3-15. *Line 172 - typo? "costs of reviewing" rather than "costs to"?*

Response: We have made this change.

R3-16. *Line 196 - typo? "analyses" plural?*

Response: We have made this change.

R3-17. *Page 10 - Fig.3 - It is difficult to discern the red-blue gradations of the points in the plot. Might a graded, multi-colour scheme be easier to discern?*

Response: We've tried some other color schemes and haven't found anything that is obviously better. The main point of the red-blue coloring is that the redder points are in the upper right, the bluer points are in the lower left, and the purplish points are in the middle. We hope the present color scheme makes this point sufficiently well.

R3-18. *Line 214 - replace (or supplement) "reviewing" with "sorting" for greater consis-*

tency?

Response: The Reviewer's point is well-taken. Our first submission was a bit cavalier about framing the central feedback loop in terms of screening and sorting vs. in terms of the review load L and the review noise σ_Y. Of course, the two are tightly coupled: journals sort manuscripts using the reviews that referees provide. Yet, we can help the reader by keeping the framing more consistent. We have rewritten the single-journal analysis extensively to do so.

R3-19. *Line 224 - "Figure 4A, B illustrates" is not a sentence since there is no object.*

Response: Respectfully, our understanding is that "illustrate" can be an intransitive verb.

R3-20. *Lines 229-234 - The explanation given is not very clear. Some tweaks are needed; e.g. "when reviewer accuracy declines as L increases" is better than "when reviewer accuracy declines with L" in this reader's opinion.*

Response: We have changed the phrase in question to read "when reviewer accuracy declines with increasing L". In general, there is a longstanding debate about whether "X increases with Y" is preferable to "X increases as Y increases", or not. We are not going to settle the debate here. Reading between the lines, we get the sense that the Reviewer appreciates that reasonable people can disagree on this count, and we appreciate the latitude to proceed as we see best fit.

R3-21. *Lines 254-256 - why is vertical proliferation outside the scope of the paper? It strikes me as a more interesting question than horizontal proliferation? Can the authors explain?*

Response: We have added text to explain that vertical proliferation is harder to accommodate; see also our response to comment R1-1. Even Müller-Itten (2021, ref. 44) had to make substantial (and to us, questionable) assumptions to find the equilibrium in a model with two vertically differentiated journals.

R3-22. *Line 298 - I struggled to understand mathematically what "proportional to (the opposite of)" means. Presumably the relationship is not an inverse proportionality, but can the authors explain? Might it simpler and clearer to reframe things in terms of reviewer burden rather than reviewer welfare?*

Response: The Reviewer's point is well-taken. Here and throughout, we have rephrased to read "the burden on reviewers is proportional to the review load L_J." We had intended that the reviewer welfare is proportional to $-L_J$, but this is awkward because the negative of the review load is an odd thing to contemplate, as the Reviewer's comment makes clear.

R3-23. *Line 334 - "writes neatly as"? Would "can be written neatly as" be more grammat-*

ically correct?

Response: Here and throughout, we have replaced "writes as" with phrasing that will be more familiar to the journal's readership.

R3-24. *Line 435 - the authors may well be correct, but the use of the word "psychic" conjured for me a group gathered around a table in a darkened room for a séance! Might "psychological" be a good substitute?*

Response: Respectfully, to the extent that we understand, "psychic cost" and "psychological cost" mean essentially the same thing and are equally suitable here, but the former is more memorable. We prefer to keep it.

R3-25. *Line 450 - revise-and-resubmit options might encourage authors to submit less polished manuscript but in my experience the rise of preprints opposes this tendency since any manuscript defects are on public display from the get-go.*

Response: The Reviewer raises an interesting point. In the absence of hard data one way or the other, though, it still seems to us that if revision is the best one can do then there is less of an incentive to polish a manuscript for its initial submission. Because we already hedge by saying that rise-and-resubmit *may* encourage authors to submit less polished manuscripts, we prefer to leave the text as is, notwithstanding a few edits for improved clarity.

R3-26. *Line 477 - "Top authors benefit from accurate reviewing and thus prefer to submit their work to journals that offer it." strikes me as a not unreasonable statement given the simplifying assumptions of the model, but what do authors know about the quality of review at different journals in reality (beyond, obviously, the extremes). Attempts to relate the model to reality would benefit from a more informed consideration of this point and citation of studies that have attempted to evaluate variation in the quality of peer review at different journals.*

Response: We thank the Reviewer for prompting us to think harder about this paragraph. In hindsight, we had gotten a bit out over our skis here by presenting a mathematical implication of the model as established fact. Because we've added some new material to the Discussion, this paragraph now seems less essential, so we've struck it.

R3-27. *Discussion section - A major weakness of the discussion of the peer review problems that are the focus of this manuscript is the fact that the authors only consider what journals might do. This narrowness of perspective arises naturally from the construction of the model which centres on the triad of authors, reviewers and readers brought together by the journal. But to give the paper greater relevance, the discussion should be widened out. How might other stakeholders - e.g. universities, learned societies, funders- contribute to solving the problem? What about the capture of peer review by commercial publishers, who have sought to increase market share through journal proliferation? I appreciate that the authors will not*

want to provide a review of all the woes of academic publishing, but some discussion of how their model might help prompt action from stakeholders beyond journals or publishers, or might justify new or more radical approaches to scholarly communication, would be valuable.

Response: Please see our response to the Editor at the beginning of this document.
